# Tularemia and plague survey in rodents in an earthquake zone in southeastern Iran

**DOI:** 10.4178/epih/e2015050

**Published:** 2015-11-17

**Authors:** Behzad Pourhossein, Saber Esmaeili, Miklós Gyuranecz, Ehsan Mostafavi

**Affiliations:** 1 Department of Virology, School of Public Health, Tehran University of Medical Sciences, Tehran, Iran; 2 Department of Epidemiology, Pasteur Institute of Iran, Tehran, Iran; 3 National Reference Laboratory for Plague, Tularemia and Q Fever, Research Centre for Emerging and Re-emerging Infectious Diseases, Pasteur Institute of Iran, Akanlu, Kabudar Ahang, Hamadan, Iran; 4 Department of Bacteriology, Faculty of Medical Sciences, Tarbiat Modares University, Tehran, Iran; 5 Institute for Veterinary Medical Research, Centre for Agricultural Research, Hungarian Academy of Sciences, Budapest, Hungary

**Keywords:** Plague, Tularemia, Earthquakes, Iran

## Abstract

**OBJECTIVES:**

Earthquakes are one the most common natural disasters that lead to increased mortality and morbidity from transmissible diseases, partially because the rodents displaced by an earthquake can lead to an increased rate of disease transmission. The aim of this study was to evaluate the prevalence of plague and tularemia in rodents in the earthquake zones in southeastern Iran.

**METHODS:**

In April 2013, a research team was dispatched to explore the possible presence of diseases in rodents displaced by a recent earthquake magnitude 7.7 around the cities of Khash and Saravan in Sistan and Baluchestan Province. Rodents were trapped near and in the earthquake zone, in a location where an outbreak of tularemia was reported in 2007. Rodent serums were tested for a serological survey using an enzyme-linked immunosorbent assay.

**RESULTS:**

In the 13 areas that were studied, nine rodents were caught over a total of 200 trap-days. Forty-eight fleas and 10 ticks were obtained from the rodents. The ticks were from the *Hyalomma* genus and the fleas were from the *Xenopsylla* genus. All the trapped rodents were *Tatera indica*. Serological results were negative for plague, but the serum agglutination test was positive for tularemia in one of the rodents. *Tatera indica* has never been previously documented to be involved in the transmission of tularemia.

**CONCLUSIONS:**

No evidence of the plague cycle was found in the rodents of the area, but evidence was found of tularemia infection in rodents, as demonstrated by a positive serological test for tularemia in one rodent.

## INTRODUCTION

Natural disasters, including floods, earthquakes, and hurricanes, occur every year throughout the world. Outbreaks of infectious diseases after natural disasters have been recorded throughout history. Examples include an outbreak of plague following an earthquake and fire in the US (1906) [1], a plague outbreak in Maharashtra after an earthquake in India (1993) [2], coccidiosis outbreaks in the US after an earthquake (1994) [3], outbreaks of pneumonia after an earthquake in Japan (1995) [4], an outbreak of pulmonary diseases and gastrointestinal diseases after the Bam earthquake in Iran (2003) [5], an outbreak of malaria after a tsunami in India (2004) [2], and a tularemia outbreak after a flood and earthquake in Turkey (2005) [6].

Earthquakes are one of the most common natural disasters, imposing heavy costs on individuals and governments, and can cause increased mortality and morbidity from infectious diseases [7]. Iran is considered one of the world’s 10 most earthquake-prone countries. This means that on average, every five years a severe earthquake, accompanied by financial losses and/or casualties, occurs in Iran [5]. After an earthquake, public health is compromised due to limited access to clean water, lack of food and malnutrition, and an increase in direct contact between humans and infected reservoir animals and vectors [8]. Additionally, after an earthquake, people overwhelm shelters. In such situations, outbreaks of vector-borne and zoonotic diseases such as malaria, plague, tularemia, and cutaneous leishmaniasis have been reported. After disasters, it is important to assess the infection status of the intermediate hosts (rodents, mosquitoes, etc.) of the diseases that are prevalent in the region [9-11].

*Yersinia pestis* is a Gram-negative bacterium that causes plague [12]. This bacterium is a major threat to human health after natural disasters. Plague outbreaks have occurred following earthquakes in San Francisco (1906), Armenia (1988), and India (1994) [13-15].

The intracellular Gram-negative bacterium *Francisella tularensis* causes tularemia. This bacterium is circulated in wildlife between small mammals and carnivores and their ectoparasites. The bacterium is highly stable in water and soil. In natural disasters where the human ecosystem is affected, such as floods and earthquakes, closer contact inevitability occurs between humans and wildlife. The use of unsafe water due to the lack of usable and appropriate water also increases the risk of this disease [16].

Fleas are the main vector of plague bacteria [13] and ticks are one of the main vectors of tularemia [17]. Plague and tularemia are associated with different pathological symptoms in rodents. Plague leads to splenomegaly, swollen lymph nodes, and swelling and congestion in the liver and spleen, which are significant indications in an autopsy [18,19]. In rodents, an infection with *F. tularensis* can cause symptoms of pyogranulomatous inflammation in the liver and spleen that can lead to mortality within three days [20].

An outbreak of plague was reported in 1906 in the province of Sistan and Baluchestan, around Sistan Lake [21]. In a study conducted in 1973 in Iran, tularemia antibodies were found in one porcupine in this province [22]. A 2011 study conducted in Sistan and Baluchestan Province found tularemia seroprevalence among butchers and slaughterhouse workers [23]. In 2007, an outbreak, first suspected to be plague, occurred in southeastern Iran in Sistan and Baluchestan Province, between the cities of Khash and Saravan (Gosht region), leading to the hospitalization of 34 people and the deaths of eight people. The outbreak was eventually determined to be tularemia [24]. In the same year, an outbreak of plague was reported approximately 100 km to the east, in the Nimruz Province of Afghanistan [25].

A magnitude 7.7 earthquake occurred on April 20, 2013 in Sistan and Baluchestan Province, in the same region where the outbreak of tularemia occurred in 2007. Due to the possibility of an outbreak of zoonotic disease, a research team from the Pasteur Institute of Iran was dispatched to this area in order to assess the presence of plague and tularemia among rodents in this region.

## MATERIALS AND METHODS

**Figure 1.**
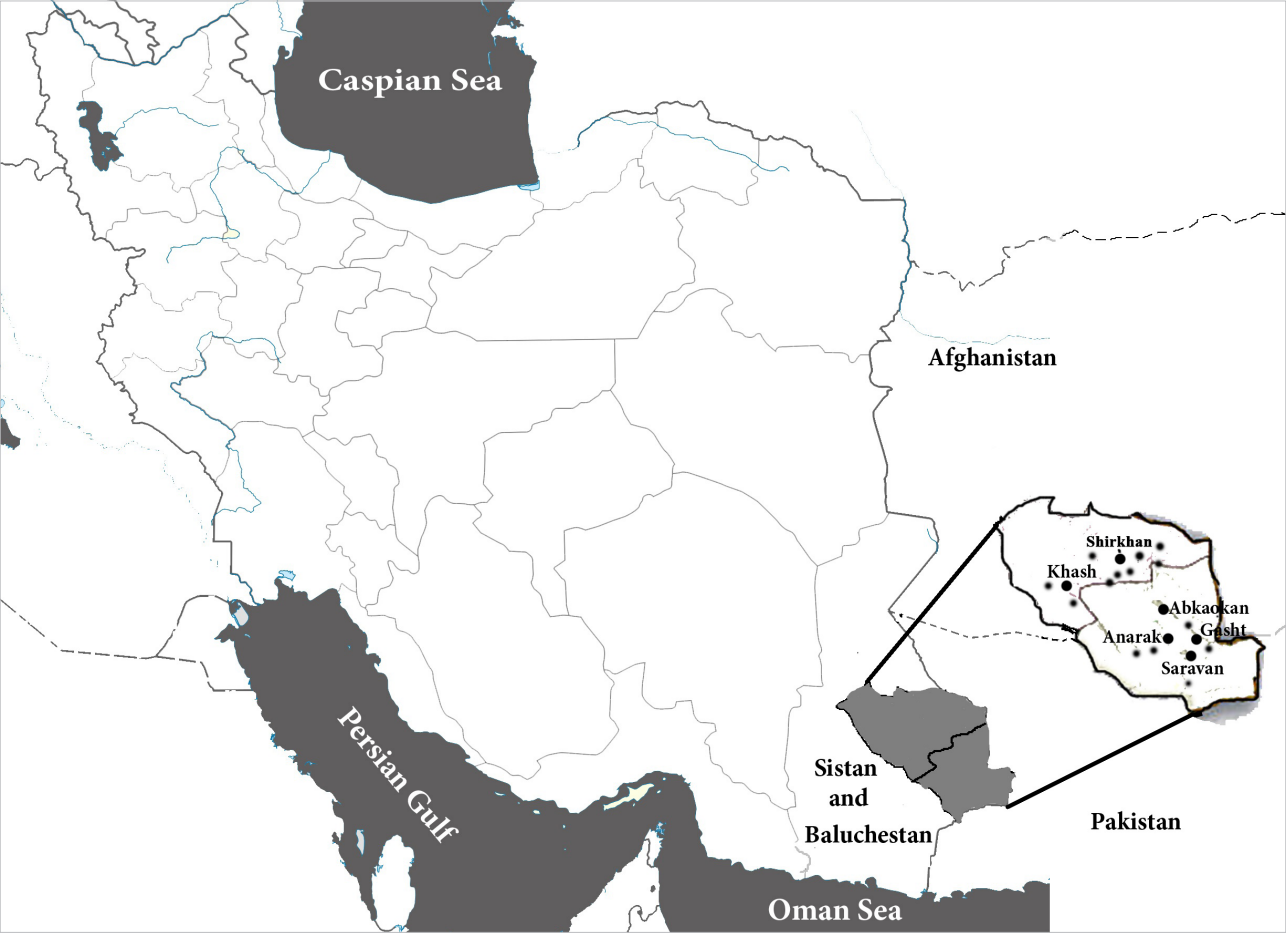
The area of study and sampling locations in Khash and Saravan in Sistan and Baluchestan Province, southeastern Iran

On April 20, 2013, at the request of the Center for Communicable Disease Control at the Ministry of Health, a Pasteur Institute of Iran rapid response team was sent to investigate the presence of plague and tularemia in the areas of Sistan and Baluchestan Province affected by the April 2013 earthquake. The research team was deployed to the area between the cities of Khash and Saravan (the area of the 2007 tularemia outbreak). Rodents were trapped in and around this region (Figure 1). Trapping for rodents was carried out around and inside of the village of Shirkhan in the Khash municipality, in Gosht, and the villages of Abkaokan and Anarak in the Saravan municipality.

The village of Shirkhan is located approximately 55 km from the center of Khash and is on the border between the metropolitan areas of Saravan and Khash. A limited number of rodent burrows were found in the vicinity of Shirkhan. Abkaokan is only approximately 15 km from Shirkhan. Rodent burrows were found around and near the village. The village of Anarak is located approximately 10 km west of Gosht (the epicenter of the earthquake), which is located 70 km north of Saravan.

Thirteen distinct areas were designated for study. Of these areas, six covered the region of the tularemia outbreak in 2007, with two sampling sites included in the village and four outside the village, in order to monitor the infection status of the rodents. Field observation, rodent trapping, and sampling of the rodents continued from April 20 to April 24, 2013 in these areas (Figure 1).

Wooden live traps with dates as bait were used to trap the rodents. Traps were checked daily and the rodents’ ectoparasites were collected on site. Traps were checked early in the morning to avoid overheating the rodents. The rodents were then transferred to the central laboratory of Saravan Health Center for blood sampling and autopsy. After chloroform anesthesia, blood samples were taken directly from the hearts of the trapped rodents and serum was obtained using centrifuges at 3,500 rpm for 10 minutes. Serum samples were stored at -20°C.

After blood samples were taken from the animals, the rodents were autopsied and lesions in various organs were assessed. The ectoparasites and rodents were preserved in alcohol and were transferred, along with the serum samples, to the Research Center for Emerging and Re-emerging Infectious Diseases (National Reference Center for Diagnosis and Research on Plague, Tularemia and Q Fever) at the Pasteur Institute of Iran. Fleas and rodents were identified according to reference standards [26-28]. Enzyme-linked immunosorbent assay (ELISA) testing was performed on the serum samples to detect immunoglobulin G (IgG) antibodies against Y. pestis according to the protocol of the World Health Organization Reference Laboratory at the Pasteur Institute of Madagascar [29]. Additionally, serological testing for tularemia was performed on rodent serum samples using the agglutination method [30].

## RESULTS

Nine rodents were caught over a total of 200 trap-days in the 13 surveyed areas, and 48 fleas and 10 ticks were isolated. All ticks were from the Ixodidae family, *Hyalomma* genus and all fleas were *Xenopsylla* spp.

On average, five fleas and one tick were visible on each rodent. All rodents were Tatera indica. Around the village of Anarak, in an area of approximately 10 km^2^, inactive nests were found, indicating a mass migration of the rodents. This may have been due to a flood and the resultant presence of transient water in riverbeds.

In the village of Kamal Abad, a porcupine (Erinaceidae) was trapped, five ticks were isolated from its surface and after blood sampling, it was sent for an autopsy to inspect its internal organs (Table 1). Pathological lesions were not observed in any of the trapped animals.

ELISA results for anti-plague antibodies (IgG) for all samples were negative. Serological testing using the agglutination method for assessing tularemia infection was positive, with serum titers of 1/80 in one rodent trapped in the Saravan municipality (Abkaokan village).

## DISCUSSION

In this study, serological tests found no cases of plague infection among the trapped rodents and porcupine, while serological testing was positive for tularemia in one rodent. The ticks and fleas that were found on the body of the trapped rodents in this study are suitable vectors for the transmission of diseases such as plague, tularemia, murine typhus, and viral diseases such as Crimean-Congo hemorrhagic fever [31-33].

Several genera of rodents, including *Apodemus* and *Rattus*, can be reservoirs of tularemia [34], but the most notable finding of this study was a serologically positive Indian gerbil (*Tatera indica*). The Indian gerbil is the most common rodent in Sistan and Baluchestan Province [22], but tularemia infection has never before been reported in this type of rodent, either in the region or in the rest of the world. It is therefore important to carry out further research on *Tatera indica* in order to establish their possible role in the transmission of tularemia.

It can be stated that the risk of tularemia transmission increased in this region after the 2013 earthquake, in light of the previous tularemia outbreak in 2007 (in Saravan and Khash in Sistan and Baluchestan Province) [24], the tularemia-positive rodent found in this study, and the changing living conditions of the human residents affected by the earthquake, who moved from homes to open spaces, resulting in close contact between humans and rodents and the consumption of unsafe water due to the lack of appropriate water sources.

No cases of human tularemia were reported following the April 2013 earthquake in this region, in contrast to the 1999 earthquake in Turkey, after which several human cases of tularemia were reported [6].

It is common for residents to fear that a natural disaster will repeat itself, especially after earthquakes. This fear leads residents to congregate outside their homes in areas without proper sanitary facilities, which can cause direct and indirect contact with rodents, increasing the likelihood of exposure to diseases transmitted by rodents [35].

As is the case for the present study, most epidemiological studies in the areas affected by natural disasters are cross-sectional surveys, meaning that they cannot show a causal link between the occurrence of disease and the disasters. Economic recessions generally occur after disasters, and resources and facilities in the affected area may be scarce. These conditions reduce the level of health, and the post-disaster period is a time when outbreaks are known to begin [36,37].

One of the limitations of this study was the lack of access to supplementary serological tests for tularemia. The agglutination test is the primary screening test for the diagnosis of tularemia, but the definitive test to detect this disease is a culture, immunofluorescence antibody, or western blot test. The agglutination test may have false-positive results because it is capable of crossreacting with several other infectious agents, such as *Brucella* spp. [38]. It would have been preferable for the positive sample be rechecked using a definitive test [39] but we had no access to such tests. In future studies of this type, it is recommended to use definitive tests to confirm the results of an initial screening.

Another limitation of this study was the low number of samples tested. During the field visit, few active rodent nests were found, whereas abandoned nests were abundant, indicating a large rodent migration due to drought, flooding, or unknown reasons over the previous years. Flooding is one of the main reasons that rodents migrate [40]. Sistan and Baluchestan Province has a hot and dry climate and steppe vegetation [40]. The earthquake in 2013 occurred during the rainy season and the team was therefore not able to trap a large number of rodents. In similar studies in the future, the research team should spend more time in the region for sampling, and sampling should be repeated two to three months after the natural disaster.

Border control in this area, with respect to the health conditions of travelers, should be considered by the relevant authorities. However, due to the presence of potential reservoirs and vectors of plague and tularemia in this region, continuous surveys are necessary to monitor these infectious diseases.

Due to the outbreak of plague in 2007 in Afghanistan and the presence of human and animal traffic between Iran and Afghanistan over the long border between the two countries, as well as the report of an outbreak of tularemia in that year (around Khash and Saravan), the Center for Communicable Disease Control should be made fully aware of endemic diseases and outbreaks in Afghanistan.

In light of the negative results for plague in this study, the positive serological result for tularemia in a rodent, and the presence of potential hosts and vectors for both diseases in this area, similar studies at other times should also be considered, and health officials, physicians, and healthcare providers need to be more sensitive to these diseases. Additionally, monitoring the movement of people living in this region, which is divided between two countries (Iran and Afghanistan), should be performed with greater precision.

## Figures and Tables

**Table 1.** The number of rodents and ectoparasites collected in Sistan and Baluchestan Province after the 2013 earthquake

Name of sampling area	Municipality	No. of locations sampled	Trap-days	Type of trapped rodents (n)	No. of ticks	No. of fleas
Shirkhan (village)	Khash	6	62	6	-	22
Abkaokan (village)	Saravan	1	15	1	-	6
Waste disposal sites	Gosht	2	28	-	-	-
Anarak (village)	Saravan	2	75	1^1^	-	-
Kamal Abad (village)	Saravan	2	20	2^2^	10	20
Total		13	200	9	10	48

1 Operator error resulted in the animal escaping.

2 One of the animals trapped was a porcupine.
